# Stem cells and exosomes: as biological agents in the diagnosis and treatment of polycystic ovary syndrome (PCOS)

**DOI:** 10.3389/fendo.2023.1269266

**Published:** 2023-10-17

**Authors:** Mahta Hadidi, Keyvan Karimabadi, Elham Ghanbari, Leila Rezakhani, Mozafar Khazaei

**Affiliations:** ^1^ Student Research Committee, Kermanshah University of Medical Sciences, Kermanshah, Iran; ^2^ Fertility and Infertility Research Center, Health Technology Institute, Kermanshah University of Medical Sciences, Kermanshah, Iran; ^3^ Department of Tissue Engineering, School of Medicine, Kermanshah University of Medical Sciences, Kermanshah, Iran

**Keywords:** polycystic ovary syndrome, exosome, stem cell, biomarker, miRNAs

## Abstract

A typical condition of the female reproductive system is polycystic ovary syndrome (PCOS). Hyperinsulinemia, insulin resistance, obesity, and hyperandrogenism are just a few of the metabolic abnormalities linked to this disease. Type 2 diabetes, hypertension, and cardiovascular disease are further issues related to PCOS. One consequence of this syndrome for which numerous treatment procedures have been developed is infertility. Metformin and clomiphene, two common allopathic medications used to treat PCOS, both have drawbacks and are ineffective. It is vital to seek novel therapeutic modalities to address these constraints. Exosomes (EXOs) are a particular class of extracellular vesicles that cells release, and they are known to play a significant role in mediating intercellular communication. A wide range of cargo, including lipids, proteins, mRNA, miRNAs, and numerous other noncoding RNAs, are contained in the nanoscale lipid bilayer exosomes. The cytokine effects of stem cells and EXOs derived from them enable the defense against metabolic diseases like PCOS. Moreover, EXO microRNAs can potentially be employed as biomarkers in the detection and management of PCOS. In this study, the potential of stem cells and exosomes are specifically investigated in the diagnosis and treatment of PCOS as one of the diseases of the female reproductive system.

## 1 Introduction

Increased androgen levels, ovulation issues, and morphological abnormalities are all symptoms of the disease known as polycystic ovarian syndrome (PCOS). The National Institutes of Health (NIH) defines PCOS as “hyperandrogenism with ovulation disorder.” This condition occurs in at least 6% to 10% of women in the fertile phase, although the frequency is suggested to be twice as high (1). The exact etiology and pathology of PCOS are not entirely known; however, a high ratio of luteinizing hormone (LH) to follicle-stimulating hormone (FSH) and an excess of gonadotropin-releasing hormone (GnRH) are recognized as its fundamental features. Evidence suggests that both internal and external factors, including genetics, epigenetics, hyperandrogenism (HA), insulin resistance (IR), and environmental factors, may be involved in the development of PCOS. It is also important to note that PCOS increases the risk of various illnesses, including type 2 diabetes, anxiety, heart disease, depression, and metabolic syndrome (2, 3). PCOS-positive women commonly exhibit low-grade chronic inflammation. Recent studies have shown that in lean, insulin-sensitive women with PCOS, the use of anti-inflammatory medication can reduce ovarian androgen release and promote ovulation. Interestingly, even in cases where insulin resistance is absent in PCOS, the data clearly indicate that inflammation acts as a crucial character in the underlying mechanism behind ovarian dysfunction (4, 5).

Mesenchymal stem cell (MSC)-based therapy bears promise as a viable therapeutic option for PCOS because of its capacity for self-renewal, differentiation potential, and immunomodulatory activities, particularly in diseases associated with inflammation. Numerous studies suggest that MSCs can potentially enhance and restore ovarian function through paracrine signaling pathways. Notably, the paracrine activity of MSCs is regulated by the RAP1/NFkB signaling pathway, which also influences immunological and inflammatory responses, making it particularly impactful on function (6). Research indicates that stem cells have the potential to improve the pathological changes associated with PCOS and reverse ovarian dysfunction. Certain pro-inflammatory cytokines like tumor necrosis factor-alpha (TNF-α), interleukin-1 beta (IL-1β), and interferon-gamma (IFN-γ) are down-regulated as part of this therapeutic effect. Additionally, fibrosis-related genes, e.g., connective tissue growth factor (CTGF), are also down-regulated to contribute to the restoration of ovarian function in females with PCOS (7, 8).

Exosomes (EXOs) are small vesicles, measuring 30 to 100 nm and protected by membrane packets, that are released by a variety of live cells under physiologically healthy or pathological circumstances. EXOs include a variety of regulatory substances, including proteins, lipids, mRNAs, and microRNAs (miRNAs). These substances have an impact on biological processes and can move between various cell types (9). These nanoparticles originate from different cells and can transport and release contents to target cells to act as intercellular mediators (10). All EXOs have membrane-associated proteins due to their endosomal origin, and these proteins can be used as biomarkers to identify EXOs. Tetraspanins, GTPases, heat-shock proteins, proteins involved in the formation of multivesicular bodies and antigen-presenting cells, and protein biomarkers including CD81, CD9, CD63, Alix, and TSG101 are some of the categories into which the biomarkers can be divided (11). Mesenchymal stem cell (MSC)-derived EXOs have recently demonstrated promise for use in treating various disorders because of their propensity to come from stem cells as well as the fact that they are more biologically stable and less immunogenic than MSCs. EXOs appear to have a therapeutic effect on female reproductive troubles, including the repair of injured endometrium, suppression of endometrial fibrosis, regulation of immunity and anti-inflammation, and inhibition of granulosa cell death in ovaries (12).

Despite the routine prescription of drugs for the treatment of PCOS, this disease remains a leading cause of female infertility in the world. New research is moving towards using the effects of stem cells and EXOs derived from them to treat this disease in the reproductive system of women. Studies have examined the effects of stem cells and EXOs on reproductive system diseases; however, the current research focuses on their potential for use specifically in the diagnosis and treatment of PCOS.

**Figure 1:**
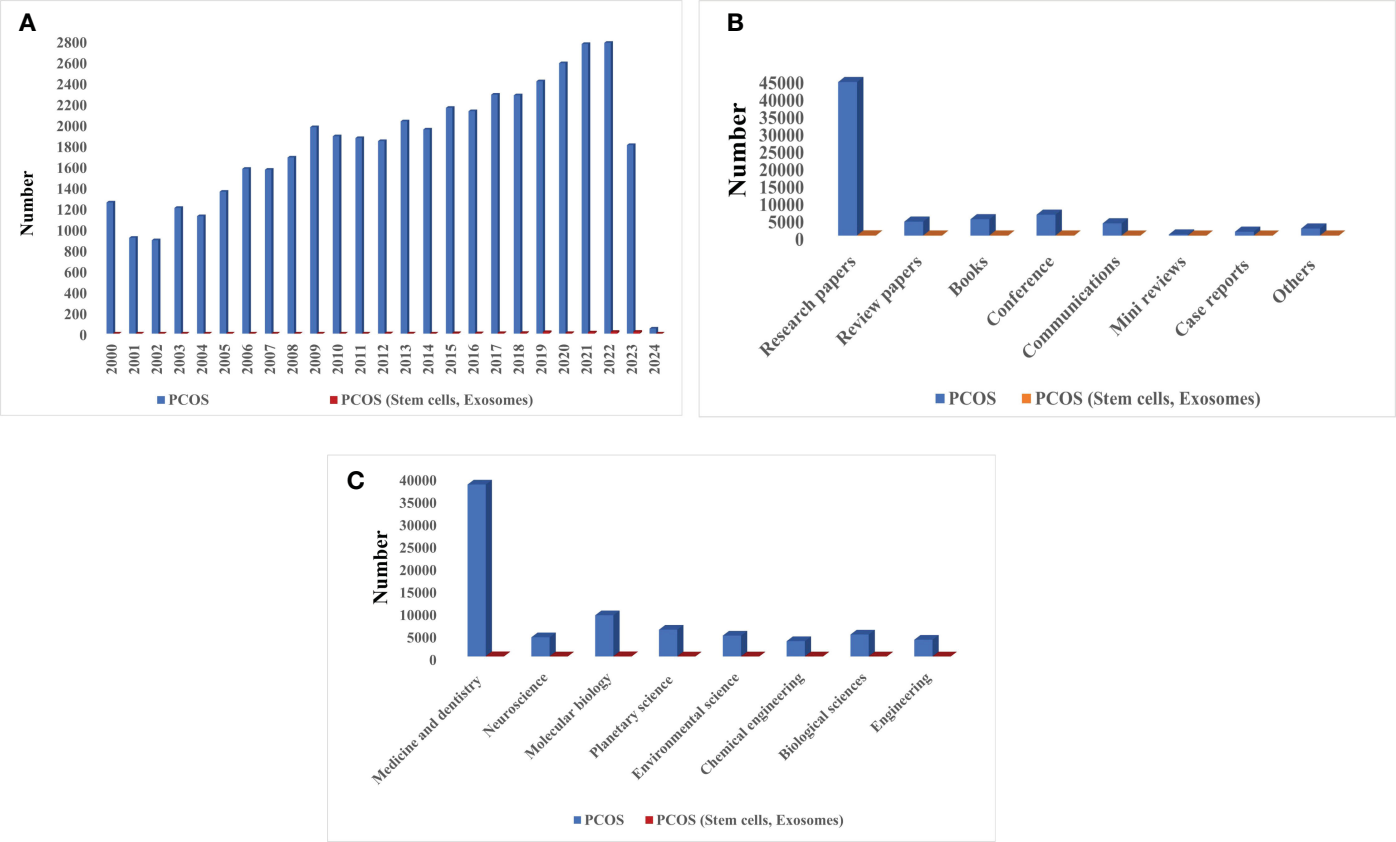
Scientific attention to PCOS based on Scopus, PubMed, and Web of Science reports: **(A)** Number of published articles on PCOS with and without stem cells and EXOs from 2000 to 2024, **(B)** Type of published literature on PCOS with and without stem cells and EXOs, **(C)** Statistics of scientific attention to different branches PCOS with and without stem cells and EXOs.

Considering the importance of fertility, it seems necessary to focus on the treatment of diseases leading to infertility such as PCOS. Studies related to this syndrome began seriously early in the 21^st^ century. Although research into the benefit of using stem cells and EXOs in diagnosing or medicating PCOS began around the same time, it had not attracted a large amount of attention among researchers. Recent years have seen the rapid development of new technologies in the field of stem cells and EXOs isolation from them; accordingly, it is hoped that these elements will be successful in the treatment of this syndrome and be applied in clinical research. **Figure 1** presents a history of studies on PCOS with and without the use of cell stem cells and their EXO derivatives.

## 2 Basics of PCOS and stem cells

PCOS is a prevalent metabolic and endocrine condition affecting women of reproductive age. It is a significant factor and the main reason for infertility among women of reproductive age (13–16). The prevalence of this heterogeneous familial disorder varies based on the population studied, but it generally ranges from 8% to 13% in adult women and around 6% in adolescent girls (17).

PCOS is a complex disease with an uncertain and specific etiology. It results from the interplay of various factors, including genetic susceptibility, intra- and extrauterine influences, as well as environmental factors (18). Some studies have reported that PCOS is characterized by chronic inflammation and an increase in cytokines such as interleukin-1β, interleukin-1 receptor antagonist, interleukin-6, interleukin-17, interleukin-18, and other factors like fibroblast growth, vascular endothelial growth, and pigment epithelium-derived factor play pathophysiological roles in the development of PCOS (14). One study showed that daughters of PCOS-afflicted mothers are also highly at risk for this disease. The factor for this predisposition is the Anti-Mullerian hormone (AMH)-coding gene, while other genes can also prove the genetic component of this disease (19, 20).

Luteinizing hormone (LH), FSH, AMH, and androgens all have an impact on the dynamic equilibrium between dormant and developing follicles in the ovary, which results in ovulation. In PCOS, the usual dynamic equilibrium breaks out. Androgen overproduction occurs in the ovary and/or the adrenal gland. The plethora of tiny follicles and incapacity to select the dominant follicle define the ovary’s morphology. Ovarian dysfunction is correlated with alterations in kisspeptin, gonadotropin-releasing hormone, LH, and FSH output. It has been emphasized how important androgen activities are to neuroendocrine function. In addition to affecting egg quality, obesity is linked to an aberrant ovarian microenvironment (17).

To diagnose PCOS in adult women, the Rotterdam PCOS criteria are commonly employed, which require the presence of two out of three findings: clinical and/or biochemical hyperandrogenism, oligo-anovulation, and polycystic ovary morphology on ultrasound. Diagnosing PCOS in adolescents, however, can be challenging because of the significant overlap of its symptoms with normal changes during puberty, making accurate diagnosis difficult most of the time (17, 18, 21). PCOS goes well beyond being considered simply as an ovarian disorder; it is now recognized as a complex, polyfactorial, polygenic, inflammatory, dysregulated steroid state autoimmune, multi-systemic disease (20).

**Figure 2:**
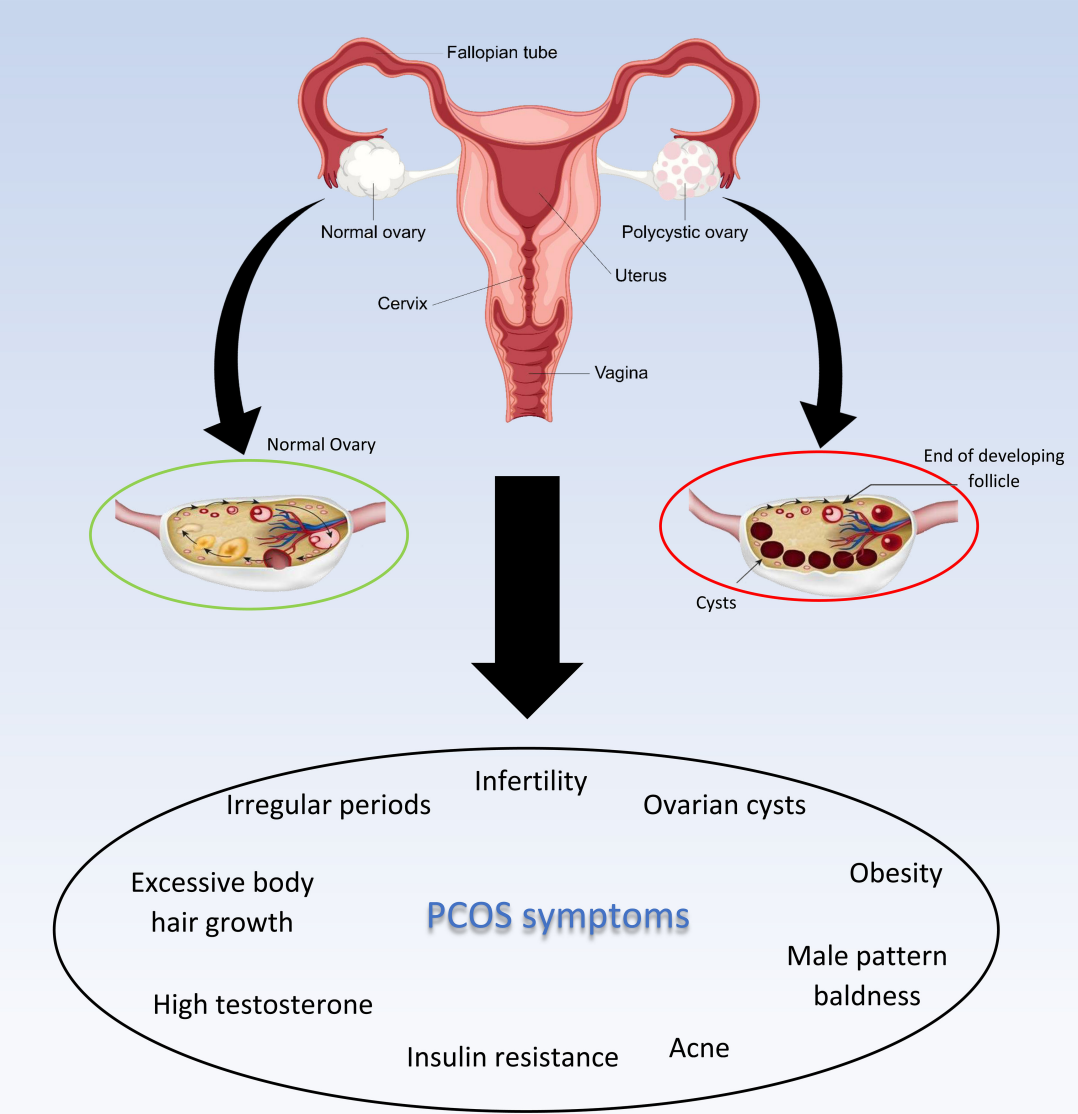
PCOS and its symptoms.

The comorbidities of PCOs can be classified into three major categories: metabolic, reproductive, and psychological. Irregular menses, subfertility, insulin resistance, obesity, cardiovascular disease, anxiety, depression, and altogether poor quality of life are the main comorbidities and consequences of PCOS (17, 18, 22). According to guidelines, the exact treatment for PCOS’s underlying cause is not yet an available option, but symptoms therapy and preventing long-term morbidity associated with PCOS are prevalent treatments these days that can be achieved by 5-10% weight loss and/or a combination of oral contraceptives and ovulation stimulating drugs (such as letrozole, clomiphene, and metformin) for women who have not resumed ovulation (**Figure 2**) (16, 18, 23). International evidence-based PCOS recommendations that stress PCOS prevention, screening, and therapy across the reproductive lifespan were created as a result of the identification of pertinent difficulties in the treatment of adolescents and women with PCOS.

Stem cells are among the most significant and vital cells in the bodies of creatures with distinctive properties. Two important and main characteristics of stem cells are their differentiation into different forms of specialized cells and their unlimited division without differentiation as long as an organism is alive (24–28). These two characteristics of stem cells are an outcome of the types of divisions they undergo. Firstly, stem cells can perform symmetric division, which leads to the production of two daughter cells, each retaining the full stem cell potential. The second kind of division is known as asymmetric divide, where one stem cell is generated, which retains its stem cell properties, and a progenitor cell is produced, which has a reduced self-renewal potential (25–29).

Stem cells are categorized based on their source into four main types: embryonic stem cells (ESCs), fetal stem cells (FSCs), adult stem cells (ASCs), and induced pluripotent stem cells (iPSCs). Each type has distinct characteristics and differentiation potentials, making them valuable for various research and medical applications (28–31). ESCs are created from the derivatives of the epiblast layer in the inner layer of the blastocyst, which can transform into the three primary germ layers of ectoderm, endoderm, and mesoderm (25, 29, 32–34).

FSCs are a category of stem cells derived from three main cell lines: fetal hematopoietic stem cells, neural stem cells (NSCs), and fetal-MSCs. These FSCs possess the ability to undergo division, proliferation, and differentiation into specialized cell types. These cell lines originate from fetal tissues and exhibit the capacity to divide, proliferate, and differentiate into specific cell types as needed (25, 29). ASCs or somatic stem cells are cell lines that exist in post-natal adult tissues and can be either uni- or pluripotent. This less differentiated cell line is the origin of the production of other types of cells and can be classified into several cell types, i.e., epidermal stem cells (EDSCs), NSCs, MSCs, and hematopoietic stem cells (HSCs) (35).

**Figure 3:**
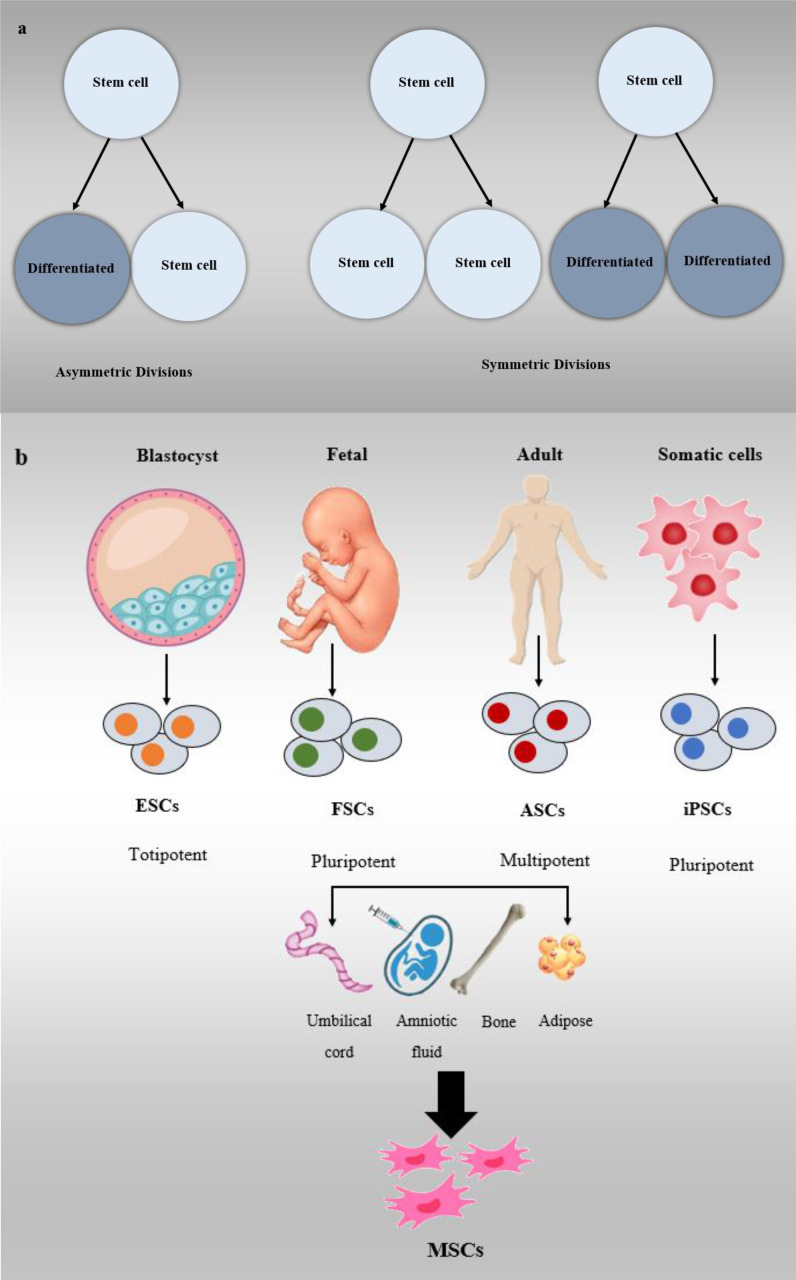
Stem cell classifications, **(A)** Symmetric and asymmetric division in stem cells, **(B)** Stem cells are divided into different types including ESCs, FSCs, ASCs, and iPSCs.

One form of stem cell that produces three differentiated primary germ layers is IPSCs, which are produced from somatic stem cells. Because they are derived from the patient’s body cells, they have a lower risk of rejection (36). Sources of MSCs include umbilical cord, bone marrow, and adipose tissue (37). Previous pre-clinical and clinical trials have shown that MSCs are effective in treating a variety of female reproductive disorders and can be used to treat PCOS (38). Other stem cell classifications are based on the ability to differentiate totipotent, pluripotent, and multipotent. The ability to transform into a differentiated cell decreases from the first category to the third category, respectively (25, 26, 28). Based on the above content and recent studies, it can be theorized that stem cells have a high capacity for the treatment of various diseases (**Figure 3**) (26, 33, 39).

## 3 Exosome: definition and application in PCOS treatment

**Figure 4:**
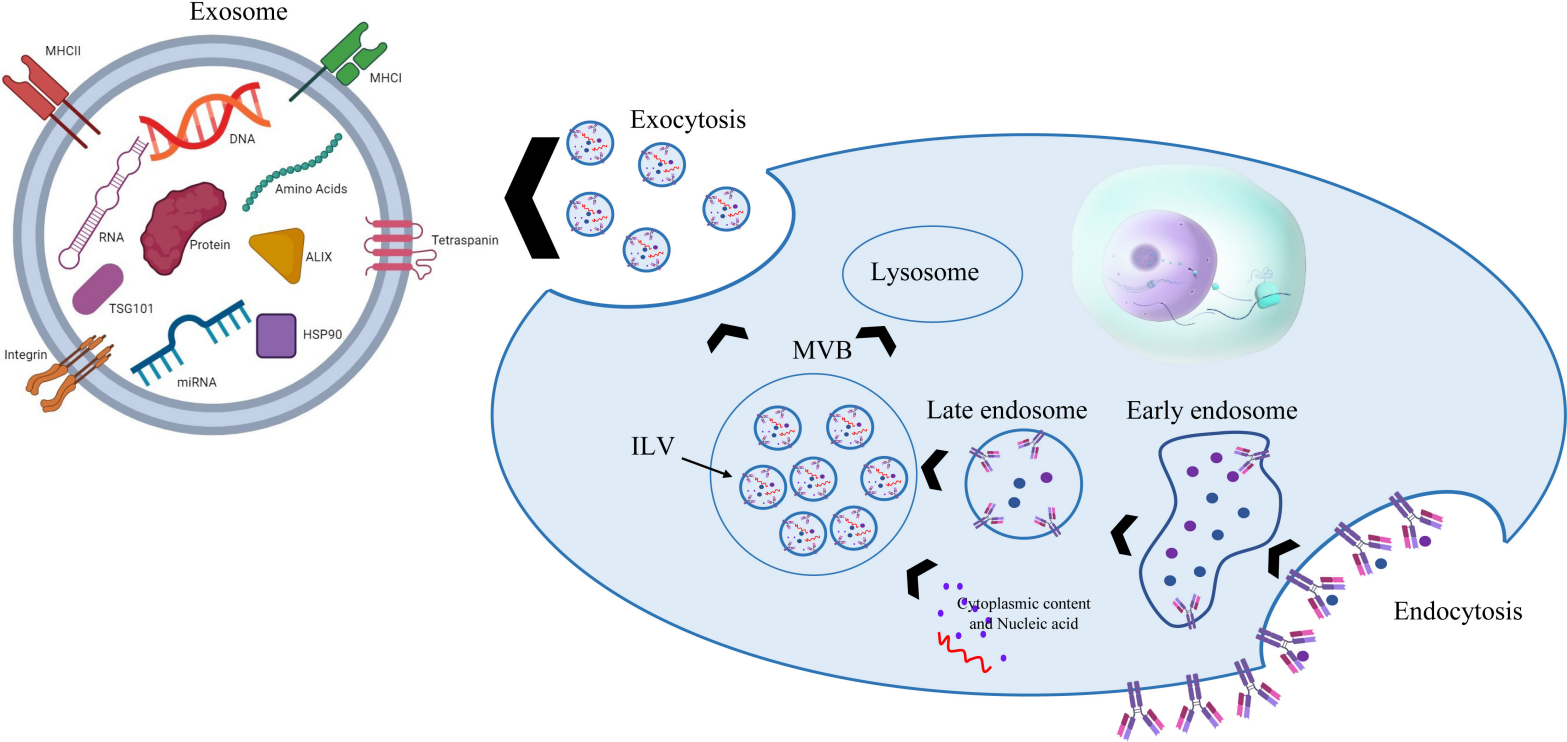
Biogenesis and structure of EXOs.

Initially, it was seen that extracellular vesicles (EVs) bud straight from the plasma membrane; however, EVs secreted by adult reticulocytes have a more intricate secretion pattern, as found in the 1980s by Pan and Harding. Cellular communication is mostly mediated by EVs, once known as cellular trash. EXOs (40 to 120 nm) are the smallest of the three EV subtypes. Johnston, a pioneer in this field, gave this EV the term “exosome” for the first time in 1987 because of its uniqueness (40, 41). EXOs are produced when multivesicular bodies (MVBs) fuse with the plasma membrane. MVBs are absorbed by the process of endocytosis, which is controlled by many processes that also govern the development of early endosomes and the inner budding of the plasma membrane. While various payloads are sorted into intraluminal vesicles (ILVs) to produce MVBs in early endosomes, a subset of proteins is returned to the plasma membrane. Finally, complete MVBs may also merge with lysosomes for degradation or with the plasma membrane to discharge ILVs as EXOs (42, 43). In recent decades, EXOs have been shown to have the potential to be used as transport vehicles and to contain several components such as miRNAs, nucleic acid RNAs, and proteins, which play important roles in extracellular communication (**Figure 4**) (11, 44).

Like MSCs, MSC-EXOs have the innate ability to suppress immune response and inflammation (45). EXOs produced by adipose MSCs have been demonstrated to impact the immune system by increasing the production of immunomodulatory cytokines, decreasing γ interferon expression and transcription factors, and limiting the local inflammatory response (46). Furthermore, MSC-derived EXOs (MSC-EXOs) exhibit an angiogenic effect by stimulating the proliferation of vascular endothelial cells. Many biological processes, including reproduction, tissue renewal and repair, and embryonic development, depend on these EXOs. Their ability to modulate these processes makes them promising candidates for various therapeutic applications, particularly in promoting tissue repair and improving reproductive outcomes (47). MSC-derived exosomes (MSC-EXOs) have the ability to transfer and regulate miRNAs and proteins, leading to the increased expression of Bcl-2 and decreased expression of Bax. Epithelial cells, cardiomyocytes, and bone cells all experience less apoptosis because of this adjustment in Bcl-2 and Bax expression. Furthermore, MSC-EXOs have a significant task in promoting tissue regeneration, aiding in the regeneration of damaged tissues (48–50).

In a rat model, AMSC-EXOs demonstrated the ability to improve fertility, treat PCOS, and protect against metabolic issues. In PCOS rats, miR-21-5p was transferred by AMSC-EXOs, which activate the IRS1/AKT pathway and improve hepatic metabolism. Additionally, they suppress the expression of the B-cell translocation gene. As a result, AMSC-EXOs ameliorate metabolic dysregulation in rats by delivering miRNAs to the liver, presenting a potential therapeutic approach for treating PCOS-related metabolic disturbances (51). AMSC-EXOs edited with miR-323-3p reduce PCOS symptoms by inhibiting cumulus cell apoptosis and increasing cell proliferation by engaging programmed cell death protein 4 (52).

According to research results, EXOs obtained from hUC-MSCs suppress chronic inflammation by decreasing the generation of inflammatory mediators such as TNF-α, and IFN-γ, elevating IL-10 levels and anti-inflammatory cytokines and reducing ovarian granulosa cell apoptosis. This research provides more proof that blocking the NF-κB signaling is the method through which anti-inflammation is accomplished (53).

According to research findings, MSC-derived exosomes are essential molecules that control androgen synthesis in an *in vitro* model and restore fertility in a mouse model of PCOS. In the context of PCOS, intravenous and intraovarian injection have both demonstrated novel therapeutic potential. In systemic regulation, such as blood sugar control, intravenous administration produces a more favorable outcome. Contrarily, intraovarian injection exhibits greater efficacy in regaining ovarian function. Although more clinical trials are required in our future research, MSC-derived exosomes may offer PCOS patients a promising therapeutic option (54).

Female rats were treated cell-free (Condition media (CM) of stem cell and exosome) after letrozole-induced PCOS. According to the study’s findings, the treatment of PCOS with both EXO and CM produced from BM-MSCs appears promising. However BM-MSCs-derived CM are superior to BM-MSCs-derived exosomes in biochemical, morphological, and functional aspects. This can be due to the fact that CM also includes numerous other biological components besides EXO (**Table 1**) (55).

**Table 1:** EXOs application in PCOS treatment.

EXOs sources	Bioactive compounds	Modeling	Finding	Ref.
UC-hMSCs	IL-10 IFN-γ TNF-α	Human	Anti-inflammatory Suppressed of NF-κB Activated signaling	(53)
AMSCs	miR-21-5p	Mice	Protect against metabolic problems, Decreased PCOS Increased fertility	(51)
AMSCs	miR-323-3p	Mice	Proliferation of cells Inhibited apoptosis in CCs through targeting PDCD4	(52)
UC-hMSCs	IL-10	*In vitro* Mice	Restored ovarian function Blood glucose control Restored fertility	(54)
BM-MSCs	IL-10 TNF-α	Rat	Restored normal histological structure of the ovaries Restored fertility	(55)

## 4 Stem cell application in PCOS treatment

MSC-based therapy has arisen as a potentially beneficial alternative for PCOS because of its immunomodulatory activities, particularly in inflammatory-related disorders, as well as its differentiation potentials and self-renewal capabilities. Extensive research has revealed the capacity of MSCs to improve and repair ovarian fertility, primarily through paracrine signaling pathways. The RAP1/NFkb signaling path acts as a crucial character in controlling MSC activity. The function of MSCs is significantly impacted by their paracrine activities, regulating inflammatory and immune responses, facilitating tissue healing, and encouraging the differentiation of progenitor cells into specialized tissue cells. According to research, women with PCOS often experience chronic inflammation; thus, they are more likely to develop type 2 diabetes (DMT2), metabolic syndrome, obesity, and insulin resistance. MSC-based therapy holds promise in addressing the underlying inflammation and restoring ovarian function, making it a potential therapeutic approach for managing PCOS and its associated complications (56).

Research findings indicate that patients with PCOS show poor results in in vitro maturation (IVM) of oocytes. However, MSCs secrete a wide variation of growth factors and cytokines that can aid in the maturation of oocytes. One study showed that the addition of 50% human bone marrow mesenchymal stromal cell-conditioned medium (hBM-MSC-CM) to the IVM medium significantly improved the fertilization rate as well as the nuclear and cytoplasmic maturation of germinal vesicle (GV) oocytes. It also led to an increase in blastocyst formation and the two-cell rate of developed oocytes from PCOS-affected mice. These findings suggest that MSC-secreted factors can positively influence the oocytes maturation in PCOS patients during in vitro procedures (57).

In animal models of PCOS, treatment with BM-MSC has shown meaningful reductions in the expression of steroidogenic genes, leading to the restoration of fertility and a decrease in inflammation. Interleukin-10 (IL-10) was discovered to be a key mediator of the therapeutic benefits of BM-MSC treatment in PCOS animals. The reported results indicate that BM-hMSC therapy effectively improves fertility and positively impacts metabolic and reproductive parameters in PCOS animal models (4).

According to studies on the causes of PCOS, inflammation and oxidative stress may contribute to the pathophysiology of the condition (58). Thus, targeting the previously mentioned systems can be an effective treatment method. As a result of their immunomodulatory, anti-oxidative, and anti-apoptotic properties, BM-MSCs are being employed in the treatment of many inflammatory illnesses (58, 59). Furthermore, it has been shown that BM-MSC transplantation is effective in the treatment of ovarian dysfunction diseases and damage (58).

PCOS patients treated with BM-MSCs showed a significant increase in the total ovary, cortex, and oocyte volumes; zona pellucida thickness; and the number of antral follicles as well as a significant decrease in the number of preantral and primary follicles compared to the PCOS ills. Moreover, the blood levels of FSH and TAC were considerably elevated, whereas the levels of LH, testosterone, and MDA and the proportion of apoptotic cells that were TUNEL-positive substantially dropped in PCOS patients treated with BM-MSCs (**Table 2**) (58).

**Table 2:** Stem cells application in PCOS treatment.

Stem cell type	Modeling	Finding	Ref.
UC-MSC	Mice	Suppression of ovarian systemic and local inflammatory responses	(38)
BM-hMSC	Mice	Decreased expression of steroidogenic gene Decreased inflammation Increased fertility	(60)
hBM-MSC-CM	Mice	Improved the oocyte-IVM, cytoplasmic development and fertilization	(57)
BM-MSCs	Mice	Increased the volume of the ovary, cortex, the number of corpuses luteum and antral follicles Decreased the number of primary and preantral follicles Decreased the serum level of testosterone, LH, MDA, and percentage of TUNEL-positive apoptotic cells	(58)
Conditioned media of BM-MSCs	Rat	Restored normal histological structure of the ovaries Restored fertility	(55)

## 5 EXO as a biomarker in PCOS

Various studies around the world have shown that different types of EXO contents, especially miRNAs, may play a role in PCOS etiology by influencing the production process and the transfer of various substances, providing the potential that they may be utilized as diagnostic and therapeutic biomarkers (61, 62).

Several experiments have investigated the function of EXOs in the context of metabolic disorders in PCOS. For instance, a study conducted in 2020 analyzed human follicular fluid (HFF) from people with and without PCOS focusing particularly on EXOs. The study revealed ten miRNAs whose expression was noticeably increased in PCOS patients (miR-6087, miR-193b-3p, miR-4745-3p, miR-199a-5p, miR-199a-3p, miR-4532, miR-629-5p, miR-199b-3p, miR-25-3p, and miR-143-3p). Conversely, these miRNAs (miR-200c-3p, miR-483-5p, miR-382-5p, miR-98-5p, miR-23b-3p, miR-200a-3p, miR-10a-5p, miR-141-3p, miR-3911, and miR-483-3p) showed a substantial reduction in expression in PCOS patients. These results present valuable insight into the potential function of EXOs and miRNAs in the metabolic alterations associated with PCOS (63).

In their study, Hu et al. focused on transfer RNAs (tRNAs) and piwi-interacting RNAs (piRNAs) in relation to PCOS pathogenesis. piRNAs are little RNA molecules that range in size from 24 to 32 nucleotides and are prevalent in the germline of all animal species. The study found different expression patterns of piRNAs when compared to controls in PCOS patients. Specifically, ten piRNAs exhibited markedly increased expression (pir-57942, pir-36441, pir-34896, pir-54998, pir-51671, pir-33221, pir-36040, pir-33226, pir-43997, and pir-33405), while ten others (pir-35414, pir-43772, pir-35413, pir-43771, pir-35469, pir-35463, pir-35468, pir-33065, pir-33387 and pir-35467) showed a substantial reduction in expression. Additionally, the authors of the study investigated tRNAs in EXOs HFF in relation to PCOS. They discovered ten tRNAs with markedly increased expression (tsrna-12363, tsrna-12365, tsrna-12364, tsrna-12362, tsrna-12360, tsrna-12361, tsrna-17099, tsrna-12359, tsrna-12395, and tsrna-17100) and ten others with substantially reduced expression (tsrna-06176, tsrna-06177, tsrna-14937, tsrna-14935, tsrna-15209, tsrna-15199, tsrna-14934, tsrna-03939, tsrna-03940 and tsrna-15198). These findings suggest that altered expression of piRNAs and tRNAs may play important mechanistic roles in PCOS pathogenesis. Moreover, previous research has associated mitochondrial tRNA mutations with PCOS, further supporting the potential significance of these findings in understanding the underlying mechanisms of PCOS (63, 64).

In an attempt to better understand how highly expressed miRNAs in follicular fluid affect steroidogenesis, particularly estradiol and progesterone secretion, Sang et al. found that 8 out of 12 miRNAs significantly influenced steroidogenesis. Estradiol levels were specifically controlled by miR-320, miR-132, miR-24, miR-520c-3p, and miR-222, whereas progesterone levels were regulated by miR-193b, miR-24, and miR-483-5p. Additionally, the target genes associated with these miRNAs were involved in cell growth and development of the immune system (65).

A 2022 study identified 157 differentially expressed miRNAs in PCOS, 33 of which were decreased, while 124 of them were substantially increased. The metabolic pathway was most affected by the differentially expressed miRNAs, showing their relevance in the pathophysiology of PCOS. The research also identified a network of miRNAs and lncRNAs connected with metabolic pathways, which shed light on the main processes driving PCOS. The study also discovered 5 miRNAs as possible indicators for PCOS diagnosis: hsa-miR-196a-3p, hsa-miR-106a-3p, hsa-miR-143-5p, hsa-miR-20a-5p, and hsa-miR-34a-5p. These miRNAs may offer interesting options for enhancing the early and accurate identification of PCOS in clinical settings (66).

In studies conducted by Wang et al. (66, 67) miR-4632, miR-146a-5p, and miR-92-5p were found to be closely associated with the incidence of PCOS. According to these studies, significant roles in the progression of PCOS may be played by long non-coding RNAs (lncRNAs), which contribute to a number of cellular functions and pathways of signaling such as the Hippo signaling pathway, endocytosis, infection with human T-lymphotropic virus type 1 (HTLV-1) and mitogen-activated protein kinases (MAPK). These results emphasize the possible uses of miRNAs and lncRNAs as treatment and diagnostic targets for PCOS and offer insightful information on the molecular processes underlying the illness (67). Researchers at Northern Jiangsu People’s Hospital examined follicular fluids produced after fertility treatment with IVF cycles and found that PCOS patients exhibited clear upregulation of certain hormone levels compared to non-PCOS patients. Particularly, blood levels of luteinizing hormone (LH), testosterone (T), estradiol (E2), anti-Mullerian hormone (AMH), and serum prolactin (PRL) were substantially greater in PCOS individuals. These results may help us learn more about PCOS and its treatment, as they provide light on the hormonal abnormalities connected to the disorder (67).

Research on the follicular fluid of women having PCOS found that out of 235 miRNAs, 29 had differing expression levels in the PCOS and control groups. In women with PCOS, the expression levels of five of these miRNAs—has-miR-9, 18b, 32, 34c, and 135a—significantly increased, suggesting that they may be involved in controlling the levels of inflammation and insulin. Additionally, three target genes (synaptotagmin1 (SYT1), insulin receptor substrate 2 (IRS2), and interleukin 8 (IL8)) were identified with significantly decreased expression in women with PCOS, which are associated to the PCOS phenotype (68).

EXOs exist in other body fluids in addition to follicular fluid; some studies have used serum samples to research the function of EXOs as PCOS biomarkers (63, 69–72). Long et al. determined that three miRNAs (miR-30c, miR-222, and miR-146a) have considerably greater production levels in patients with PCOS. Furthermore, serum insulin and miR-222 were favorably correlated, but miR-146a was inversely correlated with serum testosterone levels. Genes targeted by these miRNAs were discovered to be important in endocrine processes, apoptosis, cell cycles, metastasis, and the Jak-STAT, Wnt, and MAPK signaling pathways (73).

Another study on the serum EXOs of PCOS patients identified a relationship between PCOS and individual miRNAs, miR-126–3p, and miR-146a-5p. IL6 and tumor necrosis factor levels in the blood were discovered to be correlated with miR-146a, and these two variables are thought to be crucial in the development of PCOS (10). In another research on the microRNA profile of serum EXOs’ sequencing in women with and without PCOS, four miRNAs, namely hsa-miR-192-5p, has-miR-1299, hsa-miR-145-5p, and hsa-miR-6818-5p, were identified as potential biomarkers for PCOS. These miRNAs may hold promise for improving the diagnosis and management of PCOS because of their differential expression patterns in women with the condition compared to those without it (74).

Independent of age and body mass index (BMI), the full profiling of miRNAs in the blood of PCOS women indicated raised amounts of miR-485-3p and miR-1290 and lowered levels of miR-139-3p, miR-21-3p, miR-572, miR-361-5p, and miR-143-3p. Five miRNAs (miR-1290, -20a-5p, -139, -433, and -361-5p) were significantly related to high testosterone levels after adjusting for age and BMI. Five miRNAs were shown to be related to both abnormal glucose homeostasis and dyslipidemia particularly (miR-20a-5p, -433-3p, 1225-3p, -1290, and -361-5p) (75).

Some studies have emphasized the significance of other exosomal indications in the advancement of PCOS. For example, He et al. reported elevated miR-200c and miR-141 expressions in the granulosa lutein cells (GLCs) of PCOS patients. These two miRNAs may have crucial functions in the control of the PI3K and Wingless type protein (Wnt) signaling pathways, which may have an impact on the occurrence and development of PCOS (69, 76, 77).

In addition to the involvement of nucleic acids in PCOS, recent studies have also explored the impact of other EXO contents on the disease. Han et al. discovered that the S100-A9 protein present in EXOs, secreted by ovarian cells, granulosa cells, and leukocytes, could activate NF-κB signaling pathways within granulosa cells. This activation, in turn, led to an increase in inflammatory cytokine production and steroidogenesis disruption. These results imply that S100-A9 in EXOs may be essential for controlling granulosa cell activity and have an impact on the pathophysiology of PCOS (61, 78). Furthermore, by inhibiting DAPK1, miR-141-3p may prevent ovarian granulosa cell death and contribute to the development of PCOS (79).

Exosomal DENND1A.V2 RNA was detected in greater concentrations in PCOS patients’ urine, according to research. Additionally, PCOS theca cells expressed DENND1A.V2 protein at a greater level (80). DENND1A.V2 may impact insulin or luteinizing hormone (LH)-receptor turnover, influencing ovarian function in PCOS patients, according to another study. A component of the insulin and MAPK signaling networks, DENND1A.V2 functions as a guanine nucleotide-exchange factor for Rab, and Rab-5B, a protein associated with Ras, binds with the DENN domain. These conclusions suggest a potential role for DENND1A.V2 in the pathophysiology of PCOS through its involvement in the regulation of insulin and signaling pathways (61). According to one research, PCOS patients had substantially greater levels of mRNA expression for the proteins CYP11A, CYP19A, and HSD17b1 in their follicular fluid relative to study controls (81, 82).

Endometrial cancer, a significant concern and consequence of PCOS, has been the focus of research efforts aiming to reduce its incidence in individuals with PCOS. A recent study conducted in China investigated the levels of 55 mature miRNAs in serum EXOs of PCOS patients and found that miR-27a-5p exhibited the most significant elevation in the serum EXOs of PCOS patients. The study identified the SMAD4 gene as the target of miR-27a-5p, and its effect was shown to stimulate endometrial cancer cells to move around and invade other tissues. These findings highlight the potential role of miR-27a-5p in promoting endometrial cancer in the context of PCOS, providing valuable insights for potential therapeutic strategies to mitigate the risk of endometrial cancer in individuals with PCOS (**Table 3**) (83).

**Table 3:** Application of exosome as a biomarker in PCOs.

Model study in PCOS	EXOs source	RNA type	Ref.
HFF samples from IVF undergoing female (*in vitro*)	HFF	miRNAs/piRNAs/tRNAs	(63)
Human Serum samples (*in vitro*)	Human serum samples	miRNA	(74)
Human serum samples	Human serum samples	miRNA	(83)
HFF samples from IVF and ICSI undergoing female (*in vitro*)	HFF	miRNA	(78)
HFF (*in vitro*)	HFF	miRNA/lncRNA	(66)
HFF samples from ICSI (*in vitro*)	HFF	miRNA	(65)
HFF samples from IVF and ICSI (*in vitro*)	HFF	miRNA	(82)
HFF samples from IVF (*in vitro*)	HFF	lncRNAs	(67)
Human serum (*in vitro*)	Human serum	miRNA	(73)
Human serum (*in vitro*)	Human serum	miRNA	(10)
Granulosa cells undergoing IVF and ICSI from FF (*in vitro*)	HFF granulosa cells	miRNA	(76)
Rat ovarian granulosa cells (*in vivo*)	Rat granulosa cells	miRNA	(79)
Human Theca cells	Human Theca cells	miRNA	(80)

## Conclusion

We offer here a potentially successful avenue for the development of novel cell-based (stem cells) and cell-free (EXOs) diagnostic and therapeutic approaches for PCOS-related fertility therapy. Recent research indicates that stem cells and EXOs suppress inflammation and apoptosis, control steroidogenesis, and prevent the synthesis of androgens both *in vivo* and *in vitro*. Our knowledge of exosomal miRNA expression profiles in PCOS patients is also improved by this study. Consequently, it is worthwhile to challenge the effectiveness and efficiency of these compounds in the treatment of PCOS. We anticipate that with further developments in the study of EXOs and stem cells, these two topics will be able to significantly contribute to the early and more precise diagnosis of PCOS as well as be useful treatment and interventional tools for patients with this condition. Even though the research directions for EXOs and stem cells still need to be explored, with the persistent efforts of researchers and medical professionals, these technologies might someday become useful aids and significant medical advancements, improving the health of women with PCOS.

## Author contributions

MH: Conceptualization, Writing – original draft, Investigation. KK: Conceptualization, Investigation, Writing – original draft. EG: Conceptualization, Investigation, Writing – original draft. LR: Writing – original draft, Conceptualization, Project administration, Supervision. MK: Project administration, Supervision, Writing – review & editing.

## Glossary

**Table:**

EXO	Exosome
PCOS	Polycystic ovary syndrome
NIH	National Institutes of Health
MSCs	Mesenchymal stem cells
ASCs	Adipose stem cells
ASCs-EXOs	Adipose stem cells exosomes
ESCs	Embryonic stem cells
EDSCs	Epidermal stem cells
NSCs	Neural stem cells
HSCs	Hematopoietic stem cells
IPSCs	Induced pluripotent stem cells
EVs	Extracellular vesicles
NFkb	Nuclear factor kappa light
RAP1	Repressor activator protein 1
CM	Conditioned media
UC	Umbilical cord
PDCD4	Programmed Cell Death 4
CCs	Cumulus cells
IVM	In vitro maturation
GVs	Germinal vesicles
UC-MSCs	Umbilical cord Mesenchymal stem cells
DMT2	Type 2 diabetes mellitus
BM-hMSCs	Human bone marrow Mesenchymal stem cells
IL-10	Interleukin-10
BM-MSCs	Bone marrow Mesenchymal stem cells
BM-hMSC-CM	Human bone marrow Mesenchymal stem cell Conditioned media
TAC	Total antioxidant capacity
FSH	Follicle-stimulating hormone
LH	Luteinizing hormone
MDA	Malondialdehyde
TUNEL	Terminal deoxynucleotidyl transferase-mediated dUTP nick-end labelling
MSC-EXOs	Mesenchymal stem cell exosomes
AMSCs	Adipose mesenchymal stem cells
TNF-α	Tumor necrosis factor alpha
IFN-γ	Interferon‐gamma
UC-hMSCs	Human umbilical cord Mesenchymal stem cells
PDCD4	Programmed Cell Death 4
miRNAs	microRNAs
HFF	Human follicular fluid
piRNAs	Piwi-interacting RNA
TRNAs	Transfer RNA
LncRNAs	Long noncoding RNAs
MAPK	Mitogen-activated protein kinases
HTLV-1	Human T-lymphotropic virus type 1
T	Testosterone
PRL	Prolactin
AMH	Anti-Mullerian hormone
IL-8	Interleukin 8
IRS2	Insulin receptor substrate 2
SYT1	Synaptogamin 1
JAK-STAT	Janus kinases, signal transducer, and activator of transcription proteins STATs
AFC	Antral follicle count
GLCs	Granulosa-lutein cells
Wnt	Wingless type protein
PI3K	Phosphatidylinositol-3-kinase
DAPK1	Death-associated protein kinase 1
DENND1A.V2 RNA	DENN domain-containing 1A variant 2
CYP11A	Proteins Cytochrome P450 Family 11 Subfamily A Member 1
CYP19A	Proteins Cytochrome P450 Family 19 Subfamily A Member 1
HSD17b1	17β-Hydroxysteroid dehydrogenase 1
NIH	National Institutes of Health
LH	Luteinizing hormone
FSH	Follicle-stimulating hormone
GnRH	Gonadotropin-releasing hormone
HA	Hyperandrogenism
IR	Insulin resistance
CTGF	Connective tissue growth factor
E2	Estradiol
RAB5B	Ras-related protein Rab-5B
